# Predictors of receiving a diagnosis, referral and treatment of depression in people on antiretroviral therapy in South African primary care: a secondary analysis of data from a randomised trial

**DOI:** 10.1111/tmi.13495

**Published:** 2020-10-14

**Authors:** B. Zani, L. Fairall, I. Petersen, N. Folb, A. Bhana, G. Thornicroft, J. Hanass‐Hancock, C. Lund, M. Bachmann, the CobALT Research Team

**Affiliations:** ^1^ Knowledge Translation Unit University of Cape Town Lung Institute Cape Town South Africa; ^2^ King’s Global Health Institute King’s College London London UK; ^3^ Department of Medicine Faculty of Health Sciences University of Cape Town Cape Town South Africa; ^4^ Centre for Rural Health University of KwaZulu‐Natal Durban South Africa; ^5^ Health Systems Research Unit South African Medical Research Council Durban South Africa; ^6^ Centre for Global Mental Health King′s College London London UK; ^7^ HIV Prevention Research Unit South African Medical Research Council Durban South Africa; ^8^ School of Health Sciences University of KwaZulu‐Natal Durban South Africa; ^9^ Alan J Flisher Centre for Public Mental Health University of Cape Town Cape Town South Africa; ^10^ Norwich Medical School University of East Anglia Norwich UK

**Keywords:** antiretroviral therapy, stigma, disability, depression diagnosis, depression treatment, multimorbidity

## Abstract

**Objective:**

To describe the receipt of a diagnosis, referral and treatment for depression in people receiving antiretroviral therapy (ART), with depressive symptoms and attending primary care clinics in South Africa, and investigate factors associated with receiving these components of care.

**Methods:**

This is a secondary analysis of data from a randomised controlled trial of an intervention intended to improve detection and treatment of depression in primary care patients receiving ART. In this analysis, we combined cross‐sectional and longitudinal data from the intervention and control arms. Using regression models and adjusting for intra‐cluster correlation of outcomes, we investigated associations between socioeconomic characteristics, depressive symptoms, stress, disability and stigma, and receipt of a diagnosis, referral and treatment for depression.

**Results:**

Of 2002 participants enrolled, 18% reported a previous diagnosis of depression by a healthcare worker and 10% reported having received counselling from a specialist mental health worker. Diagnosis, referral and counselling during the follow‐up period were appropriately targeted, being independently more frequent in participants with higher enrolment scores for depressive symptoms, stress or disability. Participants with higher stigma scores at enrolment were independently less likely to receive counselling. Severe socio‐economic deprivation was common but was not associated with treatment.

**Conclusion:**

While the receipt of a diagnosis, referral and treatment for depression were uncommon, they seemed to be appropriately targeted. Socio‐economic deprivation was not associated with treatment.

## Abstract (fr)

**Objectif:**

Décrire la réception d'un diagnostic, de l’orientation et du traitement pour la dépression chez les personnes recevant un traitement antirétroviral (ART), présentant des symptômes dépressifs et fréquentant des cliniques de soins primaires en Afrique du Sud, et étudier les facteurs associés à la réception de ces éléments de soins.

**Méthodes:**

Il s'agit ici d'une analyse secondaire des données d'un essai contrôlé randomisé d'une intervention destinée à améliorer la détection et le traitement de la dépression chez les patients sous ART dans les soins primaires. Dans cette analyse, nous avons combiné les données transversales et longitudinales des bras d'intervention et témoin. En utilisant des modèles de régression et en ajustant la corrélation intra‐grappes des résultats, nous avons étudié les associations entre les caractéristiques sociodémographiques, les symptômes dépressifs, le stress, l’invalidité et la stigmatisation, et la réception d'un diagnostic, d'une orientation et d'un traitement pour la dépression.

**Résultats:**

Sur 2.002 participants inscrits, 18% ont déclaré un diagnostic antérieur de dépression par un agent de santé et 10% ont déclaré avoir reçu des conseils d'un agent spécialisé pour la santé mentale. Le diagnostic, l'orientation et le conseil pendant la période de suivi ont été ciblés de manière appropriée, étant indépendamment plus fréquents chez les participants ayant des scores d'inscription plus élevés pour les symptômes dépressifs, le stress ou l’invalidité. Les participants ayant des scores de stigmatisation plus élevés à l'inscription étaient indépendamment moins susceptibles de recevoir des conseils. La privation socioéconomique sévère était courante mais n'était pas associée au traitement.

**Conclusion:**

Bien que la réception d'un diagnostic, l’orientation et le traitement de la dépression soient rares, ils semblaient bien ciblés. La privation socioéconomique n'était pas associée au traitement.

## Introduction

Mental disorders contribute significantly to the burden of disease [1] but largely remain undiagnosed and untreated in low‐ and middle‐income countries (LMICs) [2, 3, 4, 5, 6]. Only 25% of people with common mental disorders receive treatment [5, 7]. Advances in the management of depression in primary care include newer antidepressants with improved tolerability [8, 9] and evidence‐based collaborative care strategies [10, 11, 12, 13]. Collaborative care is defined as cooperation regarding the diagnosis and/or treatment of an individual patient among two or more practitioners from different health fields [14]. These advances are important in LMICs that are short of mental health workers in primary care [15, 16, 17, 18].

With increasing multimorbidity in South Africa, and the adverse effects of depression on treatment adherence and outcomes, there is a need to investigate depression management in the growing number of people living with HIV (PLWH) and receiving antiretroviral therapy (ART) [19]. The prevalence of depression is higher among people on ART than in the general population in sub‐Saharan Africa [20, 21], which is likely to impair treatment adherence and physical health [20, 21, 22, 23]. Confounding factors such as stigma, discrimination, poverty and stressful life events that trap people, particularly women, in cycles of depression are common in PLWH [24] and may contribute to the increased prevalence of depression in ART.

Pilot intervention studies using group‐based interpersonal therapy have shown promise in reducing depression symptoms in PLWH [25, 26]. Petersen and colleagues implemented an 8‐session manualised interpersonal therapy provided by lay HIV counsellors, supported and supervised by clinical psychology trainees [25]. These sessions included depression, poverty, grief, interpersonal conflicts, social isolation, stigma and intrusive thoughts. This counselling component of the intervention was included in our collaborative care model. Nakimuli‐Mpungu and colleagues implemented an 8‐session group support psychotherapy adapted from cognitive behaviour theory, social learning theory and the sustainable livelihoods framework [26]. Counselling was provided by mental health workers with a mental health diploma or degree.

In the South African public sector, primary care is almost entirely provided by nurses, who are best placed to diagnose depression, but they need to refer diagnosed patients for counselling and initiation of antidepressant medication. Depression is however rarely diagnosed or treated [5, 7]. We carried out a pragmatic randomised trial of a health system strengthening intervention to improve diagnosis and treatment of depression among primary care patients receiving ART in South Africa [15]. The aims of the present study were to carry out a secondary analysis of trial data in order to describe the receipt of a diagnosis, referral and treatment of depression in trial participants with depressive symptoms and receiving ART, and to investigate personal and health service factors associated with receiving each component of care, which were unaffected by the trial intervention. The purpose of investigating health service factors associated with depression care is to identify favourable characteristics that could potentially be extended to other clinics, as well as possible effects of staffing and workload. The purpose of investigating patient characteristics associated with care is to assess whether care is appropriately targeted, and which patients might be relatively neglected and thus needing greater attention.

## Methods

### Study design and population

This is a secondary analysis of cross‐sectional and longitudinal data collected from the Comorbid Affective Disorders, AIDS/HIV, and Long Term Health (CobALT) study [15], combining both the intervention and control arm. The cross‐sectional data provided information of the diversity of participants’ current and past illnesses, treatment and severity of depressive symptoms, up to the time of enrolment in the study. However, these data could not clearly distinguish supposed causes from effects. The longitudinal data provided further information on changes in care and outcomes during a year of follow‐up and helped to ensure that supposed causes preceded supposed effects. CobALT was a pragmatic, cluster randomised controlled trial conducted in 40 facilities across two districts, the Dr Kenneth Kaunda and Bojanala districts in the North West province of South Africa. The trial aimed to evaluate the effectiveness of a collaborative care model for the detection and management of depression, on reducing depressive symptoms and improving viral load suppression among people on ART. Participants were 18 years or older, taking ART, with a score of 9 or more on a locally translated and validated version of the Patient Health Questionnaire‐9 (PHQ‐9) [27]. We collected data from April 2015 to October 2016 in Dr Kenneth Kaunda, and from July 2016 to January 2018 in Bojanala.

Forty facilities were equally randomised within district and sub‐district strata to intervention and control arms. Control clinics implemented the South African Department of Health’s Integrated Clinical Services Management model, which aims to reduce fragmentation of care in the context of rising multimorbidity. The model includes training primary care workers, mostly nurses, in use of the Primary Care 101 (PC101) guide—since renamed Adult Primary Care (APC)—covering communicable diseases, NCDs, women’s health and mental disorders [28]. Nurses in the control facilities were able to refer patients diagnosed with depression to primary healthcare doctors for the initiation of antidepressants as well as to mental health specialists. Limited specialist psychological and psychiatric care is also available at the district hospital. In intervention clinics, this training was supplemented with training in clinical communication skills and four additional mental health sessions to strengthen identification and management of common mental disorders, especially depression. In intervention facilities, we strengthened referral pathways for treatment by providing supplementary training of doctors around antidepressant treatment, and for counselling, by training and introducing project‐employed lay depression counsellors into intervention clinics. Counsellors received structured supervision from district‐based psychologists [15].

### Data collection and management

At enrolment, patients who volunteered participation were screened for eligibility, including PHQ‐9 screening, using an interviewer‐administered questionnaire embedded on an electronic handheld device. For those eligible and who provided written informed consent, we collected data at enrolment, and 6 and 12 months later. The questionnaire was translated from English to Setswana and Afrikaans by professional translators, and back‐translated into English to determine the accuracy of the translations. The collected data were complemented by interrogation of participants’ medical records, and a database of referrals for depression or adherence counselling provided by the lay depression counsellors. Depressive symptoms were assessed using a locally validated version of the PHQ‐9 [27, 29]. The PHQ‐9 is a nine‐item measure corresponding to the criteria upon which a diagnosis of depressive disorders is based in the Diagnostic and Statistical Manual of Mental Disorders (DSM‐IV). Items are scored based on frequency of response ranging from 0 (‘not at all’) to 3 (‘nearly every day’) and summed assuming equal weighting to yield a total score between 0 and 27. As part of the preparatory work for the trial, we validated a Setswana version of the PHQ‐9 among 676 chronic care patients in the Dr Kenneth Kaunda District, comparing the performance of a localised version of the PHQ‐9 administered by fieldworkers to that of the Structured Clinical Interview for DSM‐IV administered by a clinical psychologist. This showed that it is a valid tool for measuring depressive symptoms in the trial population [27]. However, we showed that a threshold of nine, as opposed to the more widely used threshold of 10, was more appropriate for detecting depression in this population and so applied it to the trial eligibility criteria.

At enrolment, fieldworkers administered a questionnaire covering socioeconomic and health details, diagnosis by a healthcare worker, referral, counselling and medication for depression. The receipt of a diagnosis by a healthcare worker, a doctor or a nurse, was self‐reported. In Dr Kenneth Kaunda, we asked about diagnosis by healthcare worker and when we moved to Bojanala, we added questions to ask about referrals to a doctor or psychologist. A referral only meant being sent to see another health professional, whether for medication prescription or counselling. Referrals to lay depression counsellors were obtained from the database of study counselling services and were only applicable to intervention clinics. We also collected self‐reported information on the receipt of counselling by different cadres within health services (doctor, nurse, psychologist, psychiatrist, social worker, clinic counsellor or lay depression counsellor). Participants also self‐reported depression medications being taken, and this information was supplemented by records from the clinic files. We considered receipt of counselling as receiving at least one counselling session.

The questionnaire included standardised scales locally validated and used in LMICs. The Internalised AIDS‐Related Stigma Scale assessing self‐defacing beliefs and negative perceptions in PLWH [30, 31, 32, 33], the Perceived Stress Scale measuring the degree to which life situations are considered stressful [34, 35] and the World Health Organization’s 12‐item Disability Assessment Scale which measures limitations in activity [36, 37, 38]. Interview data were uploaded and stored in a secure Microsoft SQL server at the Trial Co‐ordinating Centre at the Knowledge Translation Unit. Microsoft SQL Server is a relational database management system developed by Microsoft with the primary function of storing and retrieving data as requested by other software applications—which may run either on the same computer or on another computer across a network [39].

### Statistical methods

We first estimated the proportions of participants who were diagnosed, referred or treated for depression. Statistical analyses then investigated associations between participants’ socioeconomic indicators, health indicators and clinic characteristics as potential explanatory variables and receipt of a diagnosis, referral and treatment of depression as outcomes. Explanatory variables were reported at enrolment, and outcomes were reported at enrolment and follow‐up. We also combined outcomes recorded at either baseline or at follow‐up to indicate whether participants had ever received the respective diagnosis, referral or treatment by the end of the study. Individual health indicators used as potential explanatory variables included scores for depressive symptoms, stress, stigma and disability, and previous diagnosis of tuberculosis, hypertension and either heart attack or stroke as comorbid conditions. Clinic characteristics included the presence of a doctor at the clinic every day, medication supply available away from clinic, clinic patients per year and clinic patients per nurse per year. These clinic characteristics were investigated because they could potentially influence access to necessary treatment directly or be indirect indicators of the quality of care. Diagnosis, referral and treatment received were used as binary outcome variables. Participants who were lost to follow‐up were excluded from longitudinal analyses. We investigated baseline predictors of loss to follow‐up with logistic regression models.

In all analyses, the study’s stratified cluster sampling design was accounted for in regression models with robust adjustment for intra‐cluster correlation of outcomes, using Stata version 13.0 statistical software [40]. We used simple regression analysis to test differences in the characteristics at enrolment and at follow‐up and multiple logistic regression models retaining only those covariates that were independent predictors. Variables independently associated with the outcome in multiple logistic regression models were selected using backwards stepwise selection. At each step, explanatory variables with a *P*‐value of less than 0.10 were removed from each model [41]. The purpose of stepwise selection of explanatory variables for each model was to estimate the effects of each indicator without confounding by other characteristics. A *P*‐value of 0.05 or less was considered statistically significant. The intervention versus control arm of the randomised controlled trial was included as a covariate in all longitudinal analyses to account for the study design. Analysis of referral to project‐employed lay health counsellors was restricted to participants in intervention clinics. Regression models with outcomes reported at follow‐up included enrolment values of the same variables as covariates.

### Ethical issues

The trial was registered with ClinicalTrials.gov (NCT02407691), the Pan African Clinical Trials Registry (201504001078347) and the South African National Clinical Trials Register (DOH‐27‐0515‐5048NHREC number 4048). Ethical approval for the trial was obtained from the University of Cape Town Human Research Ethics, King’s College London Research Ethics Office and the University of Kwazulu‐Natal Biomedical Research Ethics Committee. Permission to conduct the study was granted by the North West Provincial Department of Health. All patient participants provided written informed consent.

## Results

Of the 6623 participants screened, 2002 were enrolled and interviewed at enrolment, of whom 84% were interviewed again at 12 months. Socioeconomic, health and clinic characteristics of the participants are shown in Table 1. Most of the participants were Black, women, and half were aged 42 years or older. Just under three‐quarters were unemployed, and 45% had only primary or no school education. The average monthly income was low: equivalent to US$ 72.78 in 2016 (ZAR 1040) [42]. The mean PHQ‐9 score at enrolment was 14, with 65% of participants having a score of 9–14 corresponding to mild depressive symptoms. Disability scores were high and multimorbidity common. Of 2002 participants at baseline, 327 (16.3) were not followed up. Lost to follow‐up was more likely in men and in participants with higher disability scores at baseline, but was not independently associated with any other baseline variable.

**Table 1:** Socio‐economic, psychological, health and clinic characteristics of participants with or without a previous diagnosis of depression

Characteristics	Diagnosis of depression at enrolment							Diagnosis of depression at follow‐up						
	Unadjusted estimates				Adjusted estimates			Unadjusted estimates				Adjusted estimates		
	All: n (%)	Diagnosis: n (%)	No diagnosis: n (%)	*P*‐value	aOR	95% CI	*P*‐value	All	Diagnosis: *n* (%)	No diagnosis: *n* (%)	*P*‐value	aOR	95% CI	*P*‐value
Socioeconomic characteristics	*n* = 2002	362/1997(18)	1635/1997 (82)					*n* = 1675	130/1675 (8)	1545/1675 (92)				
Age (years: mean (SD))	42 (11)	44 (10)	41 (11)	<0.001**^Ω^**			0.116		44 (10)	42 (11)	0.064			0.377
	*n* = 1997													
Male	364 (18)	58 (16)	306 (84)	0.204			0.391	294 (18)	18 (6)	276 (94)	0.285			0.202
Female	1633 (82)	304 (19)	1329 (81)					1381 (82)	112 (8)	1269 (92)				
	*n* = 1991													
Married	454 (23)	88 (19)	364 (81)	0.418			0.184	379 (23)	30 (8)	349 (92)	0.904			0.475
Not married	1537 (77)	273 (18)	1264 (82)					1295 (77)	100 (8)	1195 (92)				
Primary school education or none	891 (45)	184 (21)	707 (79)	0.022**^Ω^**			0.128	761 (45)	64 (8)	697 (92)	0.377			0.337
Secondary or tertiary education	1100 (55)	177 (16)	923 (84)					913 (55)	66 (7)	847 (93)				
Employed	531 (27)	93 (18)	438 (83)	0.706			0.772	1233 (74)	96 (8)	1137 (92)	0.969			0.694
Unemployed	1460 (73)	268 (18)	1192 (82)					441 (26)	34 (8)	407 (92)				
Total income: mean (SD) *	1.0 (1.7)	0.9 (1.1)	1.1 (1.8)	0.024**^Ω^**				1.0 (1.6)	1.0 (1.7)	1.0 (1.6)	0.814			
Psychological and health characteristics	*n* = 1991							*n* = 1674	*n* = 130	*n* = 1544				
PHQ‐score: mean (SD); N = 2002	13.8 (4.0)	15.0 (4.3)	13.5 (3.9)	<0.001**^Ω^**	1.04	1.01; 1.07	0.009**Ω**	13.8 (4.0)	14.6 (4.5)	13.7 (3.9)	0.023^Ω^			0.984
Stress score: mean (SD)	20.8 (6.6)	23.1 (6.6)	20.2 (6.5)	<0.001**^Ω^**	1.05	1.02; 1.07	<0.001^Ω^	20.7 (6.6)	23.2 (6.0)	20.5 (6.6)	<0.001^Ω^	1.03	1.01; 1.06	0.005^Ω^
Stigma score: mean (SD)	2.7 (2.1)	3.1 (2.1)	2.7 (2.1)	0.005**^Ω^**				2.7 (2.1)	3.0 (2.2)	2.7 (2.1)	0.204	1.02	1.002; 1.05	0.032^Ω^
Disability score: mean (SD)	10.3 (7.7)	12.8 (8.4)	9.8 (7.4)	<0.001**^Ω^**	1.02	>1.00; 1.04	0.027^Ω^	10.2 (7.7)	13.2 (7.9)	10.0 (7.6)	<0.001^Ω^			
Self‐reported comorbidity	*n* = 1997													
Has a hypertension diagnosis	611 (31)	146 (24)	456 (76)	0.001**^Ω^**	1.39	1.02; 1.91	0.037^Ω^	533 (32)	52 (10)	481 (90)	0.052			0.394
No hypertension diagnosis	1386 (69)	216 (16)	1170 (84)					1142 (68)	72 (9)	1064 (93)				
Previous heart attack or stroke	256 (13)	93 (36)	163 (64)	<0.001**^Ω^**	2.71	2.07; 3.56	<0.001^Ω^	230 (14)	30 (13)	200 (87)	<0.001^Ω^			0.104
No heart attack or stroke diagnosis	1741 (87)	269 (15)	1472 (85)					1445 (86)	100 (7)	1345 (93)				
Previous tuberculosis diagnosis	702 (35)	144 (21)	558 (79)	0.062			0.399	598 (36)	60 (10)	538 (90)	0.017^Ω^	1.49	0.98; 2.25	0.058
No diagnosis of tuberculosis	1295 (65)	218 (17)	1077 (83)					1077 (64)	70 (6)	1007 (94)				
History of depression at enrolment												2.80	1.95; 4.02	<0.001^Ω^
Clinic characteristics	*n* = 1997							*n* = 1675						
Daily doctor support	748 (38)	114 (15)	634 (85)	0.119			0.166	626 (37)	49 (9)	577 (92)	0.970			0.017^Ω^
No daily support	1249 (62)	248 (20)	1001 (80)					1049 (63)	81 (8)	968 (92)				
Off‐site medication collection	1204 (60)	220 (18)	984 (81)	0.906			0.925	1010 (60)	99 (10)	911 (90)	0.049^Ω^			0.204
No off‐site medication collection	793 (40)	142 (18)	651 (82)					665 (40)	31 (5)	634 (95)				
Headcount: mean (SD)^b^, ^c^	47 (23)	47 (24)	46 (21)	0.429			0.810	47 (23)	42 (20)	47 (24)	0.124			0.490
Patient‐to‐nurse ratio: mean (SD)^§^	4.1 (1.6)	4.1 (1.9)	4.2 (1.6)	0.631				4.1 (1.6)	3.6 (1.3)	4.2 (1.8)	0.038^Ω^			
Trial arm (intervention vs control)												0.52	0.26; 1.04	0.066

a Total income is a total of income received from grant, employment or business income per person. †1000 South African rand per month. *

b Headcounts: Bojanala headcount: Total headcount>=5 years Jan to June 2015; Dr Kenneth Kaunda headcount: Total headcount>=5 years April to Sep 2013. ‡

Thousands of attendances per nurse. SD = standard deviation; aOR = adjusted odds ratio; CI, confidence interval. §

c ^Ω^Indicates a statistically significant difference between groups.

### Receipt of diagnosis, referral and treatment for depression

Table 2 shows the components of depression care received by participants. At enrolment, 18% reported ever having received a diagnosis of depression by a healthcare worker, 9% reported a previous referral to a doctor or psychologist for depression, 26% reported ever receiving counselling from non‐specialist mental health services and 10% from specialist mental health services. Three people (0.2%) reported ever receiving antidepressant medication at a therapeutic dose. The proportions of those who received the respective components of care during twelve months of follow‐up were 8%, 9%, 28%, 12% and 1%.

**Table 2:** Components of depression care received: diagnosis, referral and treatment

Characteristics	At any time point		At enrolment		At follow‐up	
	*n*/*N* (%)	95% CI	*n*/*N* (%)	95% CI	*n*/*N* (%)	95% CI
Have depressive symptoms based on the PHQ‐9 sore ≥ 9	2002 (100)		2002 (100)		926/1738 (53)	51; 56
Previous diagnosis of depression	441/1997 (22)	20; 24	362/2002 (18)	16; 20	130/1675 (8)	7; 9
Referred to a doctor or psychologist for depression ^*^	155/914 (17)	17; 24	91/990 (9)	8; 13	83/909 (9)	7; 14
Referred to a doctor for depression ^*^	106/912 (12)	11; 16	60/990 (6)	5; 8	57/909 (6)	5; 9
Referred to a psychologist for depression ^*^	88/912 (10)	8; 14	56/990 (6)	5; 8	42/909 (5)	3; 7
Referred to a project‐employed lay depression counsellor ^†^	94/1008 (9)	7; 16	16/1008 (2)	1; 3	50/1002 (5)	4; 6
Received counselling from non‐mental healthcare specialist health services^§^	832/1996 (42)	39; 43	514/1996 (26)	24; 28	471/1702 (28)	25; 30
Received counselling from specialist mental health worker^¶^	377/1996 (19)	18; 29	193/1996 (10)	8; 15	225/2002 (12)	14; 18
On antidepressants at a therapeutic dose	11/2002 (1)	0; 1	3/2002 (0.2)	‐0.02; 0.32	11/1675 (1)	0; 1
Received counselling from non‐health services						
Received counselling from a traditional healer	193/1854 (10)	9; 15	121/1875 (6)	5; 8	88/1844 (5)	4; 7
Received counselling from a religious or spiritual advisor	793/1885 (42)	60; 88	490/1996 (25)	26; 41	480/1844 (26.0)	27.5; 45.0

a Data not collected in 1006 participants enrolled in Dr Kenneth Kaunda district. *

b Data obtained from interviews and counselling database and include intervention and control arm participants. †

c Non‐specialist health services refer to those services provided by general doctors or nurses. §

d Specialist mental health workers included psychiatrists, psychologists, social workers and lay depression counsellors. CI = confidence interval. ¶

### Predictors of receiving a diagnosis of depression before enrolment and at 12‐month follow‐up

Table 1 shows differences in the enrolment characteristics of participants who did or did not have a diagnosis of depression by a healthcare worker. In multivariable analysis, a history of hypertension, a previous heart attack or stroke, or higher PHQ‐9, stress and disability scores independently predicted a prior diagnosis of depression by a healthcare worker at enrolment (Table 1). Higher stress and disability scores, a diagnosis of depression by a healthcare worker at enrolment and attending clinics with off‐site medication collection independently predicted a new diagnosis of depression by a healthcare worker at 12 months (Table 1).

### Referral to a doctor, psychologist or lay depression counsellor for depression

Participants who reported referral for depression differed significantly from those who did not on several characteristics. These are shown in Tables 3 and 4. Younger age, higher stress scores and a previous heart attack or stroke were independent predictors of reporting a referral to a doctor or psychologist at enrolment (Table 3). Referrals to a doctor or psychologist were only reported in Bojanala. Referral to a doctor or psychologist at enrolment, higher enrolment disability scores and a history of tuberculosis independently predicted referrals to a doctor or psychologist at 12 months. Larger clinics were more likely to refer to a doctor or psychologist at both enrolment and follow‐up, and to a lay depression counsellor at any point, while clinics with off‐site medication collection and those with daily doctor support were less likely to refer to a doctor or psychologist at enrolment and more likely to refer to lay depression counsellors at any point (Tables 3 and 4). Older age, having secondary or tertiary education, unemployment, higher PHQ‐9 scores, attending smaller clinics or those with off‐site medication collection independently predicted referrals to lay depression counsellors (Table 4).

**Table 3:** Socio‐economic, psychological, health and clinic characteristics of participants with or without a referral to a doctor or psychologist for depression at enrolment and at follow‐up

Characteristics	Referral to a doctor or psychologist at enrolment ^*^							Referral to a doctor or psychologist during follow‐up ^*^						
	Unadjusted estimates				Adjusted estimates			Unadjusted estimates				Adjusted estimates		
	*n*	Referred *n* (%)	Not referred *n* (%)	*P*‐value	aOR	95% CI	*P*‐value	*n*	Referred *n* (%)	Not referred *n* (%)	*P*‐value	aOR	95% CI	*P*‐value
**Socioeconomic characteristics**														
Age (years): mean (SD)	990	39 (9)	40 (11)	0.191	0.98	0.96; 0.996	0.016^Ω^	787	42.5 (8.8)	41.0 (10.8)	0.100			0.127
Male	183	8 (4)	175 (96)	0.032^Ω^	2.16	0.91; 5.12	0.079	142	13 (49)	129 (91)	0.534			0.650
Female	807	83 (10)	724 (90)					645	70 (11)	575 (89)				
Married	240	18 (8)	222 (92)	0.240			0.446	187	17 (9)	170 (91)	0.486			0.526
Not married	750	73 (10)	677 (90)					600	66 (11)	534 (89)				
Primary school education or none	368	30 (8)	338 (92)	0.371			0.219	296	28 (9)	268 (91)	0.459			0.227
Secondary or tertiary education	618	61 (10)	557 (90)					491	55 (11)	436 (89)				
Employed	397	35 (9)	362 (91)	0.737			0.436	314	32 (10)	282 (90)	0.778			0.417
Unemployed	593	56 (9)	537 (91)					473	51 (11)	422 (89)				
Total income: mean (SD) ^b^, ^c^		1.0 (1.3)	1.2 (2.1)	0.427					1.0 (1.4)	1.2 (1.9)	0.372			
**Psychological characteristics**														
Enrolment PHQ‐score: mean (SD)		15.6 (3.9)	13.4 (4.0)	0.004^Ω^			0.548		14.3 (4.2)	13.3 (3.9)	0.115			0.909
Stress score: mean (SD)		22.3 (5.8)	20.8 (6.6)	0.048^Ω^	1.04	1.01; 1.07	0.014^Ω^		21.5 (6.9)	20.7 (6.6)	0.295			0.919
Stigma score: mean (SD)		2.8 (2.1)	2.8 (2.1)	0.984					2.4 (2.3)	2.8 (2.1)	0.284			
Disability score: mean (SD)		11.8 (8.0)	10.4 (7.7)	0.091			0.290		12.0 (8.8)	10.2 (7.5)	0.062	1.03	>1.00; 1.06	0.049^Ω^
Referral to a doctor or psychologist at enrolment												6.32	2.95; 13.5	<0.001^Ω^
**Comorbidity**														
Has a hypertension diagnosis	247	32 (13)	215 (87)	0.011^Ω^			0.167	212	28 (13)	184 (87)	0.104			0.902
No hypertension diagnosis	743	59 (4)	684 (96)					575	55 (10)	520 (90)				
Previous heart attack or stroke	108	19 (18)	89 (82)	0.001^Ω^	1.96	1.22; 3.16	0.005^Ω^	96	12 (13)	84 (97)	0.582			0.685
No heart attack or stroke diagnosis	882	72 (8)	810 (92)					691	71 (10)	620 (90)				
Previous tuberculosis diagnosis	287	30 (10)	257 (90)	0.418			0.962	232	35 (15)	197 (85)	<0.001^Ω^	1.46	1.05; 2.03	0.023^Ω^
No diagnosis of tuberculosis	703	61 (9)	642 (91)					555	48 (9)	507 (91)				
**Clinic characteristics**														
Daily doctor support	301	31 (10)	270 (90)	0.355			0.708	239	14 (6)	225 (94)	0.043^Ω^	0.12	0.04; 0.40	0.001^Ω^
No daily support	689	60 (9)	629 (91)					548	69 (13)	479 (87)				
Off‐site medication collection	350	30 (9)	320 (91)	0.656	0.27	0.11; 0.66	0.004^Ω^	262	29 (11)	233 (89)	0.807	0.42	0.16; 1.12	0.083
No off‐site medication collection	640	61 (10)	579 (90)					525	54 (10)	471 (90)				
Headcount: mean (SD)^d^, ^e^		54 (28)	54 (29)	0.641	1.01	1.006; 1.02	<0.001^Ω^		54 (31)	54 (29)	0.840	1.03	1.02; 1.04	<0.001^Ω^
Headcount to nurse ratio: mean (SD)^¶^		4.3 (1.6)	4.6 (1.9)	0.128					4.9 (1.9)	4.6 (1.9)	0.363			
Trial arm (intervention vs control)														0.587

a Referrals to a doctor or psychologist at enrolment, follow‐up only assessed in Bojanala district. *

b Total income is a total of income received from grant, employment or business income. †

c Reported in South African rand and per R1000. ‡

d Headcounts: Bojanala headcount: Total headcount>=5 years Jan to June 2015; Dr Kenneth Kaunda headcount: Total headcount>=5 years April to Sep 2013. §

e Thousands of attendances per nurse. SD = standard deviation; aOR = adjusted odds ratio; CI = confidence interval. ¶

f ^Ω^Indicates a statistically significant difference between groups.

**Table 4:** Socio‐economic, psychological, health and clinic characteristics of participants with or without a referral to a lay depression counsellor for depression at any point

Characteristics	Unadjusted estimates				Adjusted estimates		
	All (*n* )	Referred *n* (%)	Not referred *n* (%)	*P*‐value	aOR	95% CI	*P*‐value
**Socio‐economic characteristics**							
Age (years): mean (SD)	2002	44 (11)	41.8 (11)	0.050	1.02	>1.00; 1.03	0.045^Ω^
Male	365	15 (4)	350 (96)	0.587			0.518
Female	1637	79 (5)	1558 (95)				
Married	454	27 (6)	427 (94)	0.252	1.42	0.98; 2.07	0.065
Not married	1537	67 (4)	1470 (96)				
Primary school education or none	891	41 (5)	850 (95)	0.839	1.58	1.12; 2.21	0.009^Ω^
Secondary or tertiary education	1100	53 (5)	1047 (95)				
Employed	531	13 (2)	518 (98)	0.003^Ω^	2.27	1.12; 4.58	0.022^Ω^
Unemployed	1460	81 (6)	1379 (94)				
Total income: mean (SD) a, b		0.8 (1.0)	1.1 (1.7)	0.030^Ω^			
**Psychological characteristics**							
Enrolment PHQ‐score: mean (SD)		14.9 (4.6)	13.7 (4.0)	0.052	1.07	1.02; 1.13	0.007^Ω^
Stress score: mean (SD)		21.8 (7.3)	20.7 (6.5)	0.216			0.909
Stigma score: mean (SD)		2.5 (2.1)	2.7 (2.1)	0.287			
Disability score: mean (SD)		11.9 (8.4)	10.2 (7.7)	0.154	1.03	1.00; 1.06	0.096
**Comorbidity**							
Has a hypertension diagnosis	611	31 (5)	580 (95)	0.558			0.627
No hypertension diagnosis	1386	63 (5)	1323 (95)				
Previous heart attack or stroke	256	12 (5)	244 (95)	0.994			0.275
No heart attack or stroke diagnosis	1746	82 (5)	1664 (95)				
Previous tuberculosis diagnosis	702	42 (6)	660 (94)	0.041^Ω^			0.212
No diagnosis of tuberculosis	1295	52 (4)	1243 (96)				
Clinic characteristics							
Daily doctor support	752	32 (4)	720 (96)	0.762			0.993
No daily support	1250	62 (5)	1188 (95)				
Off‐site medication collection	1208	85 (7)	1123 (93)	<0.001^Ω^	4.51	2.51; 8.07	<0.001^Ω^
No off‐site medication collection	794	9 (1)	785 (99)				
Headcount: mean (SD)^c^, ^d^		42 (18)	47 (24)	0.175	0.98	0.97; 0.99	0.003^Ω^
Headcount‐to‐nurse ratio: mean (SD)^¶^		3.5 (1.1)	4.2 (1.6)	0.028^Ω^			

Referrals to a doctor or psychologist at enrolment, follow‐up only assessed in Bojanala district. *

a Total income is a total of income received from grant, employment or business income. †

b Reported in South African rand and per R1000. ‡

c Headcounts: Bojanala headcount: Total headcount>=5 years Jan to June 2015; Dr Kenneth Kaunda headcount: Total headcount>=5 years April to Sep 2013. §

d Thousands of attendances per nurse. SD = standard deviation; SD = standard deviation; aOR = adjusted odds ratio; CI = confidence interval. ¶

e ^Ω^Indicates a statistically significant difference between groups.

### Counselling by a specialist mental health worker for depression

People who received counselling from a specialist mental health worker and those who did not differ mostly on psychological and physical characteristics at enrolment (Tables 5 and 6). In the multivariable analysis shown in Table 5, unemployment, higher PHQ‐9 and lower stigma scores at enrolment independently predicted receiving counselling by a specialist mental health worker at any point. Older age and lower stigma scores independently predicted counselling at enrolment, while higher enrolment scores for PHQ‐9 and disability, lower scores for stigma and not having a history of hypertension independently predicted counselling at follow‐up (Table 6). Of clinic characteristics, only attending a clinic with off‐site medication collection independently predicted receiving counselling at follow‐up.

**Table 5:** Socio‐economic, psychological, health and clinic characteristics of participants who received or did not receive counselling by a specialist mental health worker at any point—unadjusted and adjusted estimates

Characteristics	Unadjusted estimates				Adjusted estimates		
	*n*	Counselling *n* (%)	No counselling *n* (%)	*P*‐value	aOR	95% CI	*P*‐value
**Socio‐economic characteristics**							
Age (years)	2002	43 (11)	42 (11)	0.054	1.01	1.00; 1.02	0.052
Male	364	75 (21)	289 (79)	0.447			
Female	1632	302 (19)	1330 (81)				
Married	454	76 (17)	378 (83)	0.212			
Not married	1542	301 (20)	1241 (80)				
Primary school education or none	891	180 (20)	711 (80)	0.362			
Secondary or tertiary education	1100	197 (18)	903 (82)				
Employed	554	85 (15)	469 (85)	0.010^Ω^	1.32	1.02; 1.71	0.035^Ω^
Unemployed	1442	292 (20)	1150 (80)				
Total income ^a^, ^b^		0.96 (1.4)	1.06 (1.78)	0.214			
**Psychological and physical characteristics**							
Enrolment PHQ‐score		14.4 (4.1)	13.6 (4.0)	0.002^Ω^	1.05	1.03; 1.08	<0.001^Ω^
Stress score		21.1 (6.5)	20.7 (6.6)	0.371			
Stigma score		2.4 (2.0)	2.8 (2.1)	<0.001^Ω^	0.89	0.84; 0.94	<0.001^Ω^
Disability score		10.9 (8.1)	10.2 (7.6)	0.316			0.429
**Comorbidities**							
Has a hypertension diagnosis	610	115 (19)	495 (81)	0.979			0.339
No hypertension diagnosis	1386	262 (19)	1124 (81)				
Previous heart attack or stroke	256	57 (22)	199 (78)	0.193			
No heart attack or stroke diagnosis	1740	320 (18)	1420 (82)				
Previous tuberculosis diagnosis	701	130 (19)	571 (81)	0.808			
No diagnosis of tuberculosis	1295	247 (19)	1048 (81)				
**Clinic characteristics**							
Daily doctor support	748	138 (18)	610 (82)	0.865			
No daily support	1248	239 (19)	1009 (81)				
Off‐site medication collection	1204	254 (21)	950 (79)	0.136			0.342
No off‐site medication collection	792	123 (16)	669 (84)				
Headcount^c^, ^d^		46 (24)	47 (23)	0.651			
Headcount‐to‐nurse ratio^§^		3.9 (1.5)	4.2 (1.7)	0.050	0.87	0.76; 0.99	0.039^Ω^

a Total income is a total of income received from grant, employment or business income. *

b Reported in South African rand and per R1000 per person per month. †

c Headcounts: Bojanala headcount: Total headcount>=5 years Jan to June 2015; Dr Kenneth Kaunda headcount: Total headcount>=5 years April to Sep 2013. ‡

d Thousands of attendances per nurse. Specialist mental health worker: psychiatrist, psychologist, social worker or lay depression counsellor; SD = standard deviation; aOR = adjusted odds ratio; CI = confidence interval. ^Ω^Indicates a statistically significant difference between groups §

**Table 6:** Socio‐economic, psychological, health and clinic characteristics of participants who received or did not receive counselling by a specialist mental health worker at enrolment and at follow‐up

	At enrolment							At follow‐up						
	Unadjusted estimates				Adjusted estimates			Unadjusted estimates				Adjusted estimates		
Characteristics	*n*	Counselling *n* (%)	No counselling *n* (%)	*P*‐value	aOR	95% CI	*P*‐value	*n*	*n* (%)	*n* (%)	*P*‐value	aOR	95% CI	*P*‐value
**Socio‐economic characteristics**														
Age (years): mean (SD)	2002	44 (11)	42 (11)	0.043^Ω^	1.02	>1.00; 1.03	0.025^Ω^	1845	43 (11)	42 (11)	0.796			0.365
Male	364	38 (10)	326 (90)	0.632				329	42 (13)	287 (87)	0.743			
Female	1632	155 (9)	1477 (91)					1516	183 (12)	1333 (88)				
Married	454	43 (9)	411 (91)	0.875				421	42 (10)	379 (90)	0.093			
Not married	1542	150 (10)	1392 (90)					1424	183 (13)	1241 (87)				
Primary school education or none	891	102 (11)	789 (89)	0.106				833	95 (11)	738 (89)	0.461			
Secondary or tertiary education	1100	91 (8)	1009 (92)					1011	130 (13)	881 (87)				
Employed	554	44 (8)	510 (92)	0.132			0.193	503	47 (9)	456 (91)	0.009^Ω^	1.34	0.99; 1.82	0.061
Unemployed	1442	149 (10)	1293 (90)					1342	178 (13)	1164 (87)				
Total income: mean (SD) ^a^, ^b^		1.05 (1.53)	1.04 (1.73)	0.933					0.90 (1.15)	1.07 (1.80)	0.031^Ω^			
**Psychological and physical characteristics**														
Enrolment PHQ‐score: mean (SD)		14.0 (3.9)	13.7 (4.0)	0.439			0.457		15.0 (4.4)	13.6 (3.9)	<0.001^Ω^	1.08	1.04; 1.12	<0.001^Ω^
Stress score: mean (SD)		20.2 (6.1)	20.8 (6.6)	0.350					21.9 (6.8)	20.5 (6.6)	0.003^Ω^			
Stigma score: mean (SD)		2.0 (2.0)	2.8 (2.1)	0.005^Ω^	0.88	0.82; 0.96	0.003^Ω^		2.4 (2.1)	2.7 (2.1)	0.045^Ω^	0.90	0.85; 0.96	0.001^Ω^
Disability score: mean (SD)		10.0 (8.2)	10.3 (7.7)	0.753			0.965		11.8 (8.1)	10.0 (7.6)	0.013^Ω^	1.02	>1.00; 1.04	0.029^Ω^
**Comorbidities**														
Has a hypertension diagnosis	610	71 (12)	539 (88)	0.133			0.431	577	61 (11)	516 (89)	0.108	0.72	0.54; 0.95	0.019^Ω^
No hypertension diagnosis	1386	122 (9)	1264 (91)					1268	164 (13)	1104 (87)				
Previous heart attack or stroke	256	33 (13)	223 (87)	0.078				243	32 (13)	221 (87)	0.616			
No heart attack or stroke diagnosis	1740	160 (9)	1580 (91)					1602	193 (12)	1402 (88)				
Previous tuberculosis diagnosis	701	64 (9)	637 (91)	0.567				649	79 (12)	570 (88)	0.984			
No diagnosis of tuberculosis	1295	129 (10)	1166 (90)					1196	146 (12)	1050 (88)				
**Clinic characteristics**														
Daily doctor support	748	76 (10)	672 (90)	0.797				698	74 (11)	624 (89)	0.403			
No daily support	1248	117 (9)	1131 (91)					1147	151 (13)	996 (87)				
Off‐site medication collection	1204	120 (10)	1084 (90)	0.795			0.994	1113	162 (15)	951 (85)	0.024^Ω^	1.87	1.15; 3.03	0.012^Ω^
No off‐site medication collection	792	73 (9)	719 (91)					732	63 (9)	669 (91)				
Headcount: mean (SD)^c^, ^d^		46 (21)	47 (24)	0.466					47 (27)	47 (23)	0.939			
Headcount‐to‐nurse ratio: mean (SD)^§^		3.8 (1.6)	4.2 (1.6)	0.109			0.131		4.0 (1.5)	4.2 (1.6)	0.290			0.776

a Total income is a total of income received from grant, employment or business income. *

b Reported in South African rand and per R1000 per person per month. †

c Headcounts: Bojanala headcount: Total headcount>=5 years Jan to June 2015; Dr Kenneth Kaunda headcount: Total headcount>=5 years April to Sep 2013. ‡

d Thousands of attendances per nurse. Specialist mental health worker: psychiatrist, psychologist, social worker or lay depression counsellor. SD = standard deviation; aOR = adjusted odds ratio; CI = confidence interval. ^Ω^Indicates a statistically significant difference between groups.§

## Discussion

This study describes the receipt of a diagnosis, referral and treatment for depression in people on ART with depressive symptoms and investigates factors associated with receiving these. One quarter of those with a PHQ‐9 score of 15–27 reported having previously been diagnosed with depression. This could be a population likely to have a long‐term risk and symptoms of depression [43]. Our findings are consistent with previous reports of the wide treatment gap for mood disorders [5, 6, 7]. Most participants reported receiving counselling from non‐specialist health services (42%), which is unsurprising given the local scarcity of specialist mental health workers [15]. Antidepressant use was very low, with only 1% on a therapeutic dose at any point during the study. This did not improve when looking at only participants with a history of depression (2%) and those with a PHQ‐9 score of 15–27 (1.5%).

Diagnosis, referral and counselling did seem to be appropriately targeted, being more frequent in participants with higher PHQ‐9, stress or disability scores. However, participants who reported more stigma, a key problem in PLWH [44], were less likely to receive counselling from a specialist mental health worker.

Profound socio‐economic deprivation was common; however, differences in deprivation indicators were not associated with receiving a diagnosis, referral or treatment for depression. Unemployed participants were more likely to be referred to a lay depression counsellor and to receive counselling in the multivariable analysis. Receipt of counselling could possibly be because employed participants were less able to take time off work for counselling while a referral for unemployed participants could point to the severity of depression. Participants with more education were more likely to be referred to a lay depression counsellor. Patients with more education have been found to be more open to discuss their condition with healthcare providers [45, 46, 47], leading to more opportunities for diagnosis and referral. It is, however, surprising that in this study we did not find a significant association between education and referral to a doctor or nurse, and between education and receiving a diagnosis by healthcare worker. In a review of mental health surveys, patients with more education used mental health services more frequently [48]. This presents a challenge for closing the treatment gap in a population such as the one included in the trial where lack of education was marked.

Even though HIV prevalence in South Africa is similar in men and women [49], 82% of participants were women. This over‐representation of women can be due to several factors, including that women are more likely to receive ART than men [50, 51]. Women tend to present with affective symptoms while men tend to self‐medicate depressive symptoms with substances [52], which is not assessed using the PHQ‐9. No clinic characteristics were consistently associated with gaps in receiving care.

This study has several limitations. Referrals to a doctor are usually for assessment and medication initiation, while referrals to a psychologist are for counselling. In this analysis, these two questions were combined as the number of participants with either of these referrals was very low. This question was only asked in Bojanala district which perhaps affected the number of responses. Referrals to lay depression counsellors were only applicable to clinics assigned to the intervention; thus, the analysis for related questions was limited to those participants.

Although the PHQ‐9 questionnaire is not used for clinical diagnosis, it gives an indication of the patients who are at risk of depression. Having not confirmed a clinical diagnosis in participants, we are unable to estimate the exact proportion of participants who should be accessing treatment.

This study highlights the need to incorporate diagnosis, referral and treatment for depression in primary care for people on ART. Until late 2018, antidepressants needed to be prescribed by doctors in South Africa. The 2018 Essential Medicines List and Standard Treatment Guidelines now make provision for nurses to initiate fluoxetine and citalopram for depression, although this needs to be followed up with a clear training and implementation plan. Authorising nurses to prescribe non‐toxic antidepressants, which are non‐fatal in overdose, and nurse training, could compensate for the lack of counselling services for patients with moderate and severe depression. This could also increase the number of patients with depression as a diagnosis, as nurses are more likely to detect a condition they are authorised to treat. Individual and group counselling was acceptable to patients [24, 25] and could be offered by lay counsellors trained in depression counselling. This cadre of healthcare worker is available but currently focus on HIV pre‐ and post‐test counselling, ART initiation counselling and adherence counselling [25]. Community health workers could be used to raise awareness about depression during household assessments, community engagement and alongside wellness campaigns. With these strategies, linkage to care for depression is likely to improve, with more people appropriately diagnosed, referred and receiving treatment.

## Conclusion

While the receipt of a diagnosis, referral and treatment for depression were uncommon, they seem to be appropriately targeted, being more frequent in participants with higher scores for depressive symptoms, stress or disability scores. Socio‐economic deprivation and comorbidity were common but were not associated with treatment.
